# Sleep and Cardio-Metabolic Disease

**DOI:** 10.1007/s11886-017-0916-0

**Published:** 2017-09-19

**Authors:** Francesco P. Cappuccio, Michelle A. Miller

**Affiliations:** 1 Warwick Medical School, Division of Health Sciences, University of Warwick, Gibbet Hill Road, Coventry, CV4 7AL UK; 2 University Hospitals Coventry and Warwickshire NHS Trust, Clifford Bridge Road, Coventry, CV2 2DX UK

**Keywords:** Sleep deprivation, Cardiovascular disease, Obesity, Diabetes, Hypertension, Naps

## Abstract

**Purpose of Review:**

This review summarises and discusses the epidemiological evidence suggesting a causal relationship between sleep duration and cardio-metabolic risk and outcomes in population.

**Recent Findings:**

Sleep duration is affected by a variety of cultural, social, psychological, behavioural, pathophysiological and environmental influences. Changes in modern society—like longer working hours, more shift-work, 24/7 availability of commodities and 24-h global connectivity—have been associated with a gradual reduction in sleep duration and sleeping patterns across westernised populations. We review the evidence of an association between sleep disturbances and the development of cardio-metabolic risk and disease and discuss the implications for causality of these associations.

**Summary:**

Prolonged curtailment of sleep duration is a risk factor for the development of obesity, diabetes, hypertension, heart disease and stroke and may contribute, in the long-term, to premature death.

## Introduction

Most creatures in the animal kingdom spend a significant amount of time asleep. Humans tend to sleep for a third of their lives. The amount required varies across the life course with more time spent in infancy and childhood, eventually settling into a pattern of 7 to 8 h per night. Sufficient sleep is necessary for optimal daytime functioning, performance and wellbeing, yet the amount of sleep that people get varies greatly.

The duration of sleep shows secular trends alongside changes in modern society that require longer hours of work, more shift work and “24/7” availability and use of commodities. These changes have reduced the average duration of sleep and modified the patterns of sleep across westernised populations, with increased reporting of fatigue, tiredness and excessive daytime sleepiness. Data published from the National Sleep Foundation suggest a significant decline in the average duration of sleep of Americans in the last 100 years, with a loss of about 1.5 h per night, from the average 9.0 h per night in 1910 to the average 7.5 h per night reported in 2014. This sleep curtailment has been attributed by many mainly to lifestyle changes.

## Sleep and Self-Reported Ill Health

Epidemiologic studies of self-reported sleep and health status began to appear about 50 years ago. However, in the last 20 years, there has been an explosion of population science on the relationship between sleep duration and a variety of health outcomes, and the implications for public health have become apparent [1••]. An exploratory study using an anonymous questionnaire probing self-reported sleep and ill-health in over 17,000 college students, aged 17–30 years, from 27 non-health-related universities from 24 countries, showed that short sleep durations (less than 7 h per night) were associated with poorer self-rated health in men and women, while longer sleep durations were not [2]. Countries with the shortest sleep duration had the worst self-rated health (all in the Far East Asia) with 30–40% ill health in Japan and South Korea and 20–30% in Thailand and Taiwan. These results clearly do not imply a causal relationship but point to a new interest in the health and social implications of sleep deprivation and the potential importance for public health.

## Short Sleep and Obesity

In the last few decades, there has been a significant increase in the prevalence of obesity worldwide and the World Health Organization has declared it a global epidemic. The rise in obesity has been paralleled by a steady constant reduction in the average sleep time, as indicated by the results of national surveys in USA in the last century [3]. The ‘inverse’ parallel trends have sparked a new interest in exploring the possible connection between these two distinct and apparently independent patterns.

### Children and Adolescents

Obesity in childhood has reached epidemic proportions in recent years and is a cause of physical and psychological problems, including low self-esteem. It often continues into adulthood, where it causes major morbidity, disability and premature death, including type 2 diabetes and cardiovascular disease. Several studies have reported associations between short sleep duration and the risk of obesity in children and adolescents, with increased estimated risk as high as 90% [4•]. The early studies have been predominantly cross-sectional, i.e. they measured duration of sleep and presence of obesity at the same time point, and therefore, whilst suggestive of possible relationships, were unable to support a cause-effect association. In other words, their results could have been consistent with two hypotheses: that a reduced duration of sleep could be the cause of obesity or, on the contrary, that obesity was the main cause of reduced sleep [5]. Both explanations are plausible. It is important, therefore, to ascertain whether the reduced duration of sleep (the exposure of interest) preceded the development of obesity (the outcome of interest). To address the temporal sequence whereby the ‘exposure’ of interest should precede the ‘outcome’ to support a link of ‘causality’, as indicated by Sir Austin Bradford Hill [6], prospective longitudinal studies in children have shown that short duration of sleep may indeed precede the development of overweight or obesity, in support of a plausible causal link between short duration of sleep and the development of obesity [7]. The potential public health implications of a causal association would be far reaching. However, whilst highly encouraging, these studies had limitations in that sleep assessment had often been based on parental reports or self-reports rather than on direct measurements, and body mass index had been the main outcome measure of obesity. More recently, however, more objective measures of sleep—using actigraphy—and more specific measures of adiposity (fat mass vs lean body mass) seem to confirm the association originally described [8].

### Adults

Short sleep is also associated with the risk of obesity in cross-sectional studies of adults (approx. 55%) [4•]. However, prospective studies are less clear in establishing the temporal sequence and further studies are still on-going [9]. One reason offered to explain why the prospective association is less clear is that with time, additional more powerful factors (physical inactivity, overfeeding) intervene, masking the effect of sleep deprivation in the determination of weight gain.

## How Would Short Duration of Sleep Cause Obesity?

There are several lines of evidence to suggest plausible mechanisms. In short-term, experiments in healthy volunteers, severe sleep deprivation causes an increase in energy intake and a reduction in energy expenditure through activation of hormonal responses that regulate appetite and energy balance [10, 11•]. During sleep deprivation, there are reciprocal changes in leptin, a hormone produced by fat cells (adipocytes) that regulate energy stores and satiety, and ghrelin, a hormone produced by the stomach that enhances appetite. These two hormones regulate hunger and satiety. In normal circumstances, when energy stores are low and the stomach is empty, leptin falls and ghrelin increases to stimulate energy intake and appetite. Conversely, when the body has accumulated enough energy through food, leptin increases and ghrelin falls. During sleep deprivation, this system is activated so that leptin is suppressed and ghrelin is stimulated, determining hunger, increased appetite and greater energy intake and storage in adipocytes with concomitant reduction in energy [10, 12]. These responses to sleep deprivation, if sustained over a longer period of time, would facilitate weight gain. Sleeping less would also give people more time to eat and to engage in other sedentary activities, as exemplified by children and adolescents who like to stay up late to play on their computer or watch TV or to interact with social networks whilst snacking [13, 14].

In principle, the associations seen between sleep deprivation and overweight or obesity is open to the possibility of a ‘reverse causality’ pathway [5], whereby obesity causes short or disrupted sleep (or both) because of breathing problems at night and the effect of inflammatory markers on the brain’s regulation of the circadian rhythm [15]. The results of controlled intervention studies in healthy volunteers, the results of prospective associations between short sleep duration and weight gain and the compelling evidence on other metabolic effects lend support to the first ‘causal hypothesis’.

## Short Sleep, Glucose Metabolism and Diabetes

Type 2 diabetes (the commonest form of diabetes in the world) is a chronic condition characterised by the inability of the body to utilise glucose from the circulation (glucose intolerance) with the result of high circulating levels of glucose in the bloodstream (hyperglycaemia). This is caused by either a resistance of the peripheral tissues (especially muscle) to the action of the hormone insulin to take up glucose into the cell (insulin resistance) or to the lack of appropriate production of insulin from the pancreas in response to a glucose load. One of the most important risk factors for the development of type 2 diabetes is overweight or obesity. The latter is characterised by an increasing degree of glucose intolerance due to insulin resistance, eventually leading to overt type 2 diabetes. Diabetes is one of the most powerful, though preventable, causes of cardiovascular diseases (i.e. coronary heart disease, stroke and kidney disease).

### Short Sleep and Glucose Metabolism

Short-term, acute, laboratory and cross-sectional observational studies indicate that disturbed or reduced sleep is associated with glucose intolerance, insulin resistance, reduced acute insulin response to glucose and a reduction in the disposition index, thus predisposing individuals to type 2 diabetes [11•]. Moreover, the same metabolic dysregulations can be observed in association with disruptions in sleep quality [11•]. These acute observations are fully reversible when sleep duration is restored. Therefore, the effects of acute, short-term sleep deprivation or disruption are reversible. Yet, many individuals believe they adapt to chronic sleep loss as easily or that recovery requires only a single extended sleep episode. This is not the case. The cumulative detrimental effects of sustained and prolonged chronic sleep loss (deprivation) are not reversed, so that prolonged life-style habits of sleep curtailment may lead to long-term adverse health and safety consequences [16]. Finally, prolonged sleep restriction with concurrent circadian disruption decreases resting metabolic rate and increases post-prandial plasma glucose (from inadequate insulin secretion) [17]. So, sleeping less and at the wrong time of the circadian cycle has compounding negative effects on glucose and insulin metabolism.

This area of research has produced convincing evidence of the direct influence of sleep on metabolic, as well as genetic, pathways. In the first clinical study linking sleep restriction to an alteration of a molecular metabolic pathway, the authors studied seven healthy volunteers in a randomised crossover trail of 4 days of sleep deprivation (4.5 h per night) and 4 days of normal sleep (8.5 h per night). They monitored the stages of sleep with polysomnography and adherence to bed-time with actigraphy. Caloric intake and meals were kept constant throughout the study. At the end of each period, the participants underwent an intravenous glucose tolerance test (IVGTT)—to measure total body insulin sensitivity—and a subcutaneous abdominal fat biopsy to isolate adipocytes. The researchers then exposed the adipocytes ‘in vitro’ to incremental insulin concentrations to measure the ability of insulin to increase the phosphorylation of Akt, an important step in the insulin-signalling pathway [18••]. The results show that sleep deprivation is associated with a 30% reduction in the phosphorylation of Akt, indicating reduced peripheral insulin response. This was paralleled by an expected reduction in total body insulin sensitivity. These results substantially challenge the traditional views that the primary purpose of sleep is confined to restorative effects on the central nervous system, pointing to a much wider influence of sleep on bodily functions, including metabolism, adipose tissue, cardiovascular function and possibly more [19].

To further this concept, there is evidence, using gene transcriptome analysis, that sleep restriction can up- and down-regulate the expression of genes mainly associated with not only circadian rhythms and sleep homeostasis (rather expected), but also with those involved in oxidative stress and metabolism [20•].

### Short Sleep and Diabetes

From the experimental data discussed, we can summarise as follows: in *short-term*, *acute*, laboratory and cross-sectional studies, disturbed or reduced sleep is associated with glucose intolerance, insulin resistance, reduced acute insulin response to glucose and a reduction in the disposition index, reduced peripheral insulin response and up- and down-regulation of the expression of genes involved in metabolic pathways, all predisposing factors to the development of type 2 diabetes.

It is, therefore, obvious to ask the question: does sleep deprivation predict the risk of developing type 2 diabetes? The causality of the association, the generalizability of the results and their extrapolation to *longer-term* effects of *sustained* sleep disturbances, however, will require the verification that short sleepers have a greater risk of developing type 2 diabetes in prospective population studies to establish a temporal sequence between exposure and outcome (excluding reverse causality).

The aggregate analysis of prospective longitudinal studies carried out in populations around the world supports the concept that individuals who sleep less than 6 h per night have a 28% greater risk of developing type 2 diabetes compared to those sleeping 6–8 h per night [21•]. The risk is even greater when disrupted quality of sleep is considered (as difficulty in initiating or maintaining sleep), with risks estimated to rise to 57 and 84%, respectively. A recent systematic review and meta-analysis of 36 studies involving over 1 million participants compared the risk associated with sleep disturbances and diabetes with that associated with other traditional risk factors, like overweight, family history and physical inactivity [22]. The analysis showed that the risk of developing type 2 diabetes in people with sustained sleep deprivation was comparable to that attributable to other well-known cardio-metabolic risk factors.

## Short Sleep and Hypertension

### What Is Hypertension?

High blood pressure (or hypertension) is a leading global health risk [23]. It is the largest known risk factor for cardiovascular disease (coronary heart disease, stroke, kidney disease and vascular dementia) and related disability. Often described as ‘silent killer’ because it rarely causes symptoms, high blood pressure had a global age-standardised prevalence of 24% (1 in 4) in men and 20% (1 in 5) in women in 2015 [23]. The number of adults with hypertension increased from 594 million in 1975 to 1.13 billion in 2015, with the increase largely seen in low- and middle-income countries [23].

In high-income countries, like the USA and the UK, about half of the adult population do not know what their blood pressure is. Of those with hypertension, more than 50% are unaware of it, and of those who take treatment, only a third shows good blood pressure control. In low- and middle-income countries, the picture is much worse [24].

Up to 80% of premature death from cardiovascular disease can be prevented through better public health. Whilst some risk factors (age, ethnicity, gender, genetics) cannot be modified, addressing modifiable risk factors will yield significant health and economic gains. Reducing excessive dietary salt, improving poor diet and reducing obesity, avoiding excessive alcohol consumption, increasing level of physical activity and improving socio-economic conditions and mental health are all cost-effective public health interventions.

When these actions are not sufficient, drug therapy is used to reduce blood pressure and the burden of cardiovascular complications.

### Role of Sleep

In physiological conditions, our blood pressure follows a diurnal pattern with a fall at night whilst we rest and sleep (referred as ‘nocturnal dip’). This fall is due to a variety of mechanisms, including supine position, muscle relaxation and reduced sympathetic tone. However, in recent years, it has become apparent that many individuals may not present the expected nocturnal dip in blood pressure (‘non-dippers’). This phenomenon may present both in those with normal ‘day-time’ blood pressure as well as in those with high ‘day-time’ blood pressure. Non-dippers have a higher risk of developing cardiovascular disease compared to dippers [25].

**Fig. 1:**
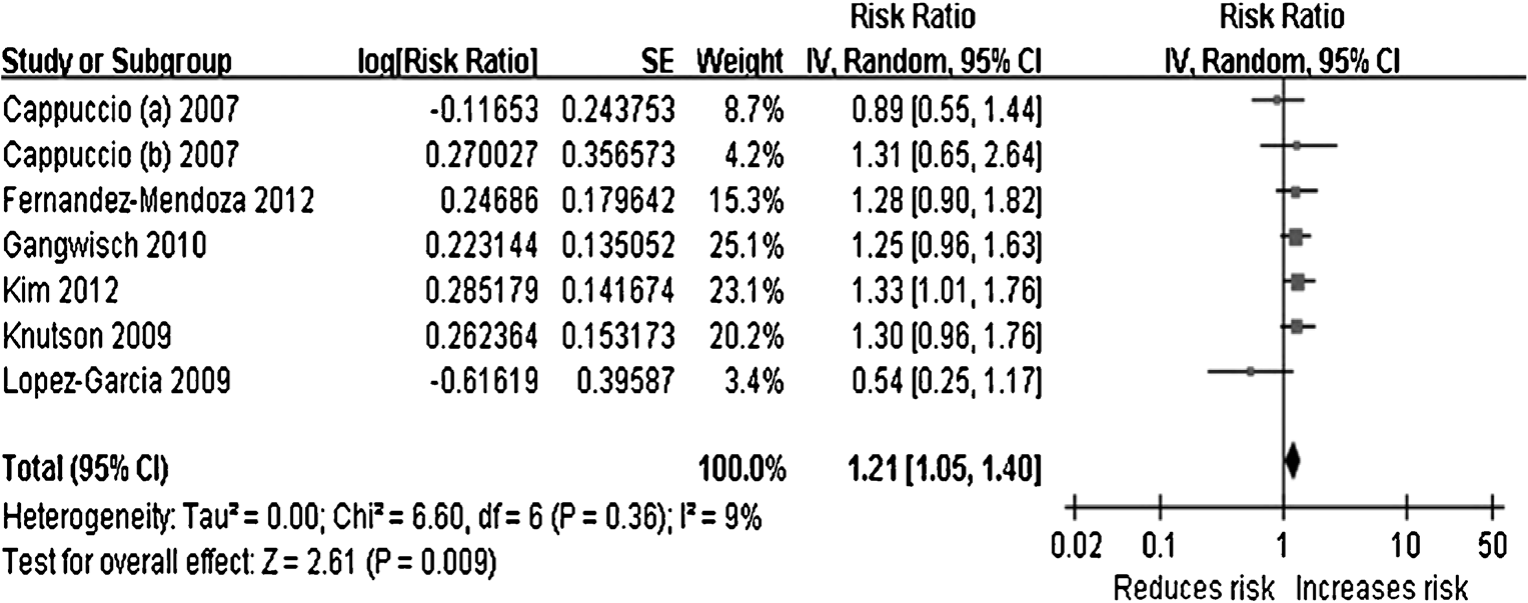
Forest plots of the prospective associations between short duration of sleep and the incidence of hypertension in population studies in adults*. *Results are reported as risk ratio and 95% confidence intervals. (Adapted by permission from Macmillan Publishers Ltd.: Meng L et al. Hypertens Res 2013; 36: 985–95) [28]

Early findings from British and American studies suggest associations between sleep duration and hypertension risk. Specifically, cross-sectional analyses showed a significant, consistent association between short sleep duration (< 5 h per night) and risk of hypertension. In some studies, the association was stronger among women [26], which was attenuated in prospective analyses after multivariate adjustment [27]. A recent review of the available studies estimates the risk of developing hypertension in short sleepers at 21% [28] (Fig. 1). The effect of sleep deprivation on the incidence of hypertension is detected also independently of the severity of sleep-breathing problems (as measured by the apnoea-hypopnoea index or AHI) [29]. The effect is detectable early in childhood and adolescence affecting both day-time and night-time blood pressure [30], suggesting that sleep disturbances not only raise night blood pressure by disrupting sleep but exert prolonged carryover effects on day-time blood pressure leading to hypertension. Finally, in a group of elderly men studied with polysomnography and followed-up for many years, it has been established that short sleep is an independent predictor of the development of hypertension predominantly because of a significant reduction in slow-wave sleep, the restorative stage of sleep [31].

An important step in epidemiological research to imply causality is the evidence of ‘reversibility’, i.e. the evidence that if one is able to modify the risk factor, the outcome should be modified in the direction of the observed relationship. One of the main limitations in this field of research is the absence of a standard validated tool for extending sleep (without the use of hypnotics or other pharmacological agents) in a sustained manner. Sleep extension can be achieved in a variety of ways, but it invariably will involve multiple behavioural modifications aided by somewhat structural changes in the environment that surrounds us and that exert pressure on our time and the opportunities we have to engage in ‘sleep’. One encouraging experiment was carried out with success in 22 participants with stage 1 hypertension who were randomised to either a 6-week period aimed to increase bedtime by 1 h daily (sleep extension group) or to a 6-week period (sleep maintenance group) aiming to maintain habitual bedtime [32]. During the trial, the sleep extension group increased the sleep duration (directly measured by actigraphy) by an average of 35 min per night compared to the sleep maintenance group. At the same time, their blood pressure measured throughout the 24 h was reduced by an additional 7/4 mmHg by the end of the interventions, suggesting a direct beneficial effect of sleep extension on blood pressure.

## Short Sleep and Other Conditions

### Lipids

Blood lipid profiles characterised by high total cholesterol and LDL-cholesterol, high triglycerides and/or low HDL-cholesterol are well-established risk factors for cardiovascular disease. They are also more commonly associated with other metabolic abnormalities like obesity, insulin resistance and diabetes. Some longitudinal evidence suggests an association between short duration of sleep and an unfavourable pattern of blood lipid profile, particularly detectable in adolescents [33]. However, a recent review of all the available evidence pooled together, whilst suggesting a trend for a 10% greater risk of developing hypercholesterolaemia in those sleeping 5 h or less, does not provide unequivocal evidence for an independent effect of sleep deprivation on lipid metabolism [34].

### Vascular Calcifications

The concept of a possible causal link between sleep deprivation and cardio-metabolic risk is strengthened by the evidence obtained from the Coronary Artery Risk Development in Young Adults (CARDIA) cohort in Chicago. In this study, 18- to 30-year-old black and white men and women were studied and followed-up for 5 years. At baseline, sleep metrics (wrist actigraphy measured duration and fragmentation, daytime sleepiness, overall quality, self-reported duration) were examined and a CT scan of their heart was carried out to measure calcifications of their coronary arteries. A similar CT scan was repeated 5 years later. After excluding the confounding effect of a variety of behavioural, life-style and metabolic factors, participants who slept 5 h or less had a sharp increase in the risk of developing coronary calcifications, risk factors for myocardial infarction (i.e. heart attacks). The rate of development of calcification was 33% greater for every hour of sleep curtailment [35]. The effect was twice as strong in women as in men, although the reason for this gender difference is not known.

## Short Sleep and Health Outcomes

From what we have seen so far, it is apparent that sleep deprivation—whether short- or long-term—is strongly and consistently associated with a variety of biological mechanisms (hormonal changes in regulation of energy balance and appetite, activation of inflammatory markers, glucose intolerance and insulin resistance, molecular effects in adipocytes, up- and down-regulation of gene expression, vascular calcifications) and risk factors (overweight and obesity, type-2 diabetes, hypertension) that collectively are strong predictors of the likelihood to develop cardiovascular disease. If these associations were to reflect cause-effect relationships, we would expect to find that those who have long-term sleep deprivation (i.e. those who regularly sleep less than the average for the population) would be more likely to develop cardiovascular disease (like coronary heart disease and stroke) and die more often from it [36].

### Coronary Heart Disease

In large population studies, people have been asked about their sleeping habits and have been followed up for many years, often decades, and their health outcomes have been recorded carefully over the years. When these studies have been put together and analysed, it has become apparent that, despite the variability between studies due to different populations, various methods of assessments etc., those who were reporting shorter duration of sleep (usually less than 5 or 6 h per night) were more likely to experience episode of coronary heart disease and to die from it when compared to those usually sleeping on average 6 to 8 h per night. The early studies indicate an average increase in risk of 48% [37••]. The risk of developing coronary heart disease is further enhanced if the short sleep is also associated to poor quality sleep [38], suggesting an independent role of quantity as well as quality of sleep on cardiovascular risk.

### Stroke

Similar to what was seen for coronary heart disease, short sleepers display a greater risk of developing fatal and non-fatal strokes over a long term [37], equivalent to an approximate 15% higher risk [39].

#### Mortality

One of the most surprising result of the epidemiological studies exploring the relationships between short duration of sleep and health outcomes is the description of a 12% increased risk of all-cause mortality in short sleepers [40••]. This finding has been consistently confirmed in independent analyses from different researchers and in different countries. These results, if attributable to a direct cause-effect, would reinforce the general concept that sleep is such a fundamental function in human biology that a sustained habit of insufficient sleep (currently estimated as less than 5 to 6 h per day on a regular basis) would be a likely contributor of premature death. It is of interest to highlight that a careful study in a representative British population has established that the higher risk of mortality that is seen in people who tend to curtail their average sleeping time is due mainly to an increase in cardiovascular mortality rather than non-cardiovascular mortality [41•].

**Fig. 2:**
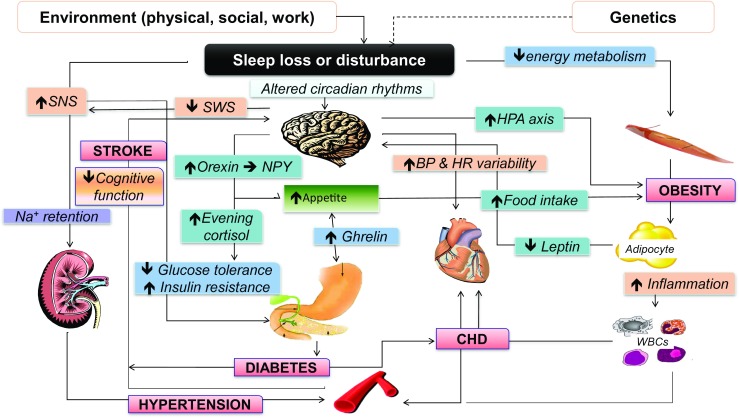
Possible mechanistic pathways linking short duration of sleep and adverse cardiovascular health. *BP* blood pressure, *HbA1c* haemoglobin A1c, *HDL* high-density lipoprotein cholesterol, *HR* heart rate, *LDL* low-density lipoprotein cholesterol, *PAI-1* plasminogen activator inhibitor-1, *SNS* sympathetic nervous system, *SWS* slow-wave sleep, *Trigs* triglycerides. (Adapted with permission from Miller MA, Cappuccio FP. J Hum Hypert 2013; 27: 583–588) [42]

In summary, Fig. 2 puts together the current state of knowledge that links sleep deprivation and health outcomes [42]. It is immediately apparent that the traditional belief that sleep pertains exclusively to the brain is being challenged by recent evidence to suggest a much wider control that sleep exerts on several organs and systems, from the heart to the kidney, the metabolic pathways to adipose tissue to the vasculature. The concept that sleep is an idle state of a suspended mind closer to a state of death must be abandoned. Sleep is an active physiological process greatly needed by our body to sustain vital functions, in the short as well as in the longer term. Its trading for extra wakefulness is not without consequences.

## Napping and Risk

A long-established practice in Mediterranean areas, daytime napping—commonly known as ‘siesta’—is often associated with good health through a postulated stress relief mechanism. However, daytime sleepiness, often characterised by daytime napping, has been regarded as an early sign or risk indicator of a range of health problems, like depression, cognitive impairment, Parkinson disease and other chronic debilitating illnesses. Whilst early evidence in Mediterranean countries suggested that ‘siesta’ would be associated with favourable cardiovascular outcomes [43], the contemporary association between daytime napping and mortality risk is uncertain. Most previous studies have come from Mediterranean countries, in which the incidence of and the rate of death from cardiovascular disease tend to be low. Several prospective studies from Israel showed that an increased mortality rate was associated with siesta among older people [44], whereas a study from Greece suggested that siesta is protective against heart-related deaths, especially in working men [45]. In addition, conflicting USA-based research has found an increased risk of death associated with daytime napping in both men and women [46]. Only naps of long durations were found to be significant. In Japan, a study suggested that daytime napping was associated with increased risk of all-cause and cardiovascular death, but this association was largely explained by concomitant comorbidity [47]. Clearly, the population evidence so far is inconsistent and it may largely depend on differences in cultural, environmental and demographic factors. It remains therefore unclear whether daytime napping is beneficial or a risk factor for or marker of ill health.

A recent programme of research on the effects of daytime napping on health outcomes was completed in a representative sample of British men and women. This programme was carried out in the EPIC-Norfolk prospective cohort study, in which over 16,000 participants provided information on their sleeping patterns, including sleep duration, time in bed and daytime napping. They were then followed-up for up to 13 years [48]. After allowing for potential known confounders, daytime napping was significantly associated with an increased risk of dying [49•]. For naps of less than 1 h, the risk was increased by an average 14%, whereas for naps longer than 1 h, the risk increased further to an average 32% (Table 1). The risk was also more pronounced for deaths from respiratory diseases and in individuals older than 65 years [49•]. In the same study, daytime napping has been associated with an increased risk of developing type-2 diabetes (2.5-fold higher), especially when associated with short duration of sleep (< 6 h per night) [50•]. Finally, a similar increased risk of chronic respiratory disease was detected among shorter (< 1 h) and longer (≥ 1 h) naps, with a dose-dependent effect of 52 and 72% increased risk, respectively [51]. Comprehensive systematic reviews confirm these results [52•, 53•].

**Table 1:** Summary table of the prospective associations between day-time napping and risk of disease and death in the EPIC-Norfolk population cohort of men and women^a^

Outcome	Groups	Sample size	Relative risk	95% C.I.
Type-2 diabetes [48]	No napping	9613	Ref	
	Napping	3851	1.30	1.01–1.69
Respiratory disease [49•]	No napping	7878	Ref	
	Nap < 1 h	2831	1.32	1.15–1.52
	Nap ≥ 1 h	269	1.54	1.14–2.09
Chronic lower respiratory disease [49•]	No napping	7878	Ref	
	Nap < 1 h	2831	1.52	1.18–1.96
	Nap ≥ 1 h	269	1.72	1.01–2.92
All-cause mortality [47]	No napping	11,493	Ref	
	Nap < 1 h	4412	1.14	1.02–1.27
	Nap ≥ 1 h	469	1.32	1.04–1.68

^a^Results are expressed as relative risk (95% confidence intervals) between napping and reference category

## Long Sleep Duration and Ill Health

It is important to mention that the associations between duration of sleep and health outcomes, whether morbid or fatal, show clear-cut and consistent U-shaped curves with the lowest risk usually seen between 6 and 8 h per night and increased risks also detectable for ‘long’ duration of sleep (> 8 h per night) [21•, 27, 37••, 39, 40••, 41•]. However, the evidence does not fulfil all the necessary criteria for implying causality (in particular absence of plausible biological mechanisms), the current interpretation is that long duration of sleep is rather a marker or a symptom of pre-clinical ill health. However, further research is needed.

## Conclusions

There is a strong and consistent association between short duration of sleep and cardio-metabolic risk factors and outcomes. The associations may reflect causality [6] as the effects are strong (large relative risks); they are consistent (confirmed in different populations for several end-points), they show a temporal sequence (short sleep preceding end-points), there is a consistent threshold of effects, there are several potential plausible biological mechanisms involved (genetic, molecular, cellular, physiological), and the reversibility of the effect is confirmed when tested in controlled trial conditions (at least in the short-term). Day-time napping and long duration of sleep are also associated with ill health and worse health outcomes. These associations are more likely to reflect the fact that napping and long sleep may be markers of compensation of sleep deprivation (forced day-time napping) or of sub-clinical chronic and/or debilitating ill health.
